# The Brain Medicine Clinic: two cases highlighting the advantages of integrative care

**DOI:** 10.1192/bjo.2022.631

**Published:** 2023-05-25

**Authors:** Seyyedeh Fatemeh Bahari Saravi, Sara Berman Mitchell, Sarah Levitt

**Affiliations:** Division of Neurology, Department of Medicine, Sunnybrook Health Sciences Centre, Toronto, Ontario, Canada; and Department of Medicine, Temerty Faculty of Medicine, University of Toronto, Toronto, Ontario, Canada; Division of Neurology, Department of Medicine, Sunnybrook Health Sciences Centre, Toronto, Ontario, Canada; and Neurology Quality and Innovation Lab, University of Toronto, Toronto, Ontario, Canada; Centre for Mental Health, University Health Network, Toronto, Ontario, Canada; and Department of Psychiatry, University of Toronto, Toronto, Ontario, Canada

**Keywords:** Clinical neurology, dementia, psychological testing, psychosocial interventions, out-patient treatment

## Abstract

**Background:**

Current assessment and management models often do not adequately address the many aspects of managing complex brain disorders involving disordered affect, behaviour and cognition (ABC). A more collaborative model of care, where several specialties can jointly assess and manage patients with complex brain disorders, is gaining attention.

**Aims:**

In this case report, we present two cases that highlight the benefits of the ‘brain medicine’ clinical model.

**Method:**

The Brain Medicine Clinic employs an integrated clinical model in which psychiatrists and neurologists provide integrated interdisciplinary assessments of patients with complex brain disorders, leading to comprehensive assessment. We describe the clinical model and the trajectories of two patients with complex brain disorders seen in this clinic. In these case descriptions, we explain how the brain medicine clinical approach leads to an improved patient experience.

**Results:**

The Brain Medicine Clinic assessments resulted in a neurobiopsychosocial formulation of symptoms and, consequently, holistic individualised treatment plans for two patients with complex brain disorders. This approach to patients’ conditions emerges from the understanding that there are multifactorial causes of brain disorders at the social, cultural, psychological and biological level.

**Conclusions:**

Integrated interdisciplinary assessments allow for tailored treatment plans for individuals experiencing complex brain disorders, while creating efficiencies for the patient and the healthcare system.

Over the course of the 20th century, brain disorders increasingly fell into the purview of different ‘brain’ specialists, such as neurologists, neurosurgeons or psychiatrists.^1^ As psychoanalytic theories gained traction and neurologists became increasingly invested in anatomical explanations for disorders, classification schemes (e.g. ICD-10) and medical training came to reflect the idea that the assessment and management of brain disorders should be divided among specialists. This attitude has persisted, despite increasing evidence that comorbid ‘neurological’ and ‘psychiatric’ symptoms are the norm, rather than the exception.^2,3^ Many of the core psychiatric disorders – such as schizophrenia and affective disorders – can be induced by structural brain disease, whereas neurological conditions such as Huntington's disease or the behavioural variant of frontotemporal dementia can primarily manifest with psychiatric symptoms.^4,5^ Furthermore, there is increased recognition that brain disorders have multifactorial aetiologies, which increases the need for interdisciplinary teams to work collaboratively for the benefit of these patients.^4,6,7^

Shared healthcare is an approach to patient care that uses the skills and knowledge of a range of health professionals to enhance the assessment and management of health conditions.^8^ In this model, team members work collaboratively to provide holistic care and increase patient access to specialist consultation.^9,10^ In recent years, increased attention has been paid to shared care models including primary care with psychiatry and neurology specialists.^9,11^ However, there are few clinical models in which ‘brain’ specialists work side by side in an integrated fashion. Collaboration between ‘brain’ specialists has potential to improve overall patient care, including timely detection, treatment and follow-up of patients with complex brain disorders. Most importantly, concurrent consultation allows for integrated practical recommendations in real time.^12,13^

In this case report, we present two cases that introduce and demonstrate the utility of an innovative clinical model referred to as the ‘Brain Medicine Clinic’. This clinic aims to expand on shared care models for individuals experiencing complex brain disorders, defined as conditions that include symptoms involving affect, behaviour and cognition (ABC).^a^ Brain medicine is defined as a clinical programme in which healthcare providers recognise that symptoms involving ABC all stem from pathology in the same organ: the brain. Consequently, practitioners of the brain medicine approach provide integrated management plans to address such symptoms, in contrast to the dichotomised approach to neurological and psychiatric symptoms that arose during the 20th century. The Brain Medicine Clinic, established at Sunnybrook Health Sciences Centre in Toronto, Canada, aims to provide patients experiencing complex brain disorders with a ‘one-stop shop’, so that they receive timely diagnosis, symptom control and support with optimal healthcare service utilisation and navigation.

## Case reports

### Ethics statement

The authors assert that all procedures contributing to this work comply with the ethical standards of the relevant national and institutional committees on human experimentation and with the Helsinki Declaration of 1975, as revised in 2008. Informed written consent was obtained from both patients described in this case report. As per our Research Ethics Board, descriptive case reports are exempt from formal review and approval. We have omitted certain details of each case to maintain confidentiality of the participants.

### Case 1

A 55-year-old right-handed woman was assessed in the Brain Medicine Clinic for a 3-year history of cognitive impairment. The referring physician was concerned about a diagnosis of early-onset dementia, as the patient was complaining of subjective difficulties in short-term memory, word finding, attention and concentration. She had a medical history of systemic lupus erythematosus (SLE), right-sided hearing impairment and right-sided retinal surgery. The onset of cognitive symptoms occurred at the same time as her initial SLE diagnosis. Her psychiatric history was significant for panic attacks and anxiety, diagnosed on immigration to Canada from Iran in 2016. The patient continued to suffer from unprovoked panic attacks several times a week when seen in initial consultation in the Brain Medicine Clinic. A psychiatric review of systems revealed ongoing overwhelming worry, especially about the meaning of her cognitive symptoms. Her family history was significant for late-onset Alzheimer's disease in her maternal grandmother at the age of 87.

The patient's medications included alprazolam 0.25 mg for panic attacks for the previous 6 months, and prednisone 2 mg and azathioprine 125 mg daily for the previous 3 years to help manage her SLE. The patient had also been prescribed citalopram 20 mg daily for anxiety for the past 5 years. However, she had not been taking the antidepressant regularly for the few months preceding her clinic visit because she would forget. She was independent in all her instrumental and basic activities of daily living. It was noted that she required more time for multitasking and planning, which was accompanied by anxiety owing to difficulty in managing her tasks.

Mental status examination revealed an anxious and dysphoric mood with an absence of hallucinations, delusions or suicidal or homicidal ideation. She had good insight and judgement. Her cognitive testing revealed a Mini-Mental State Examination (MMSE) score of 29/30, losing one point for delayed recall, and a Montreal Cognitive Assessment (MoCA) score of 28/30, with impairments in delayed recall and fluency. A neurological examination was performed and was found to be unremarkable. Magnetic resonance imaging (MRI) of the brain without gadolinium did not show any acute pathology or significant parenchymal atrophy pattern, and there were minimal microangiopathic changes noted.

In co-consultation with a neurologist and psychiatrist in the Brain Medicine Clinic, the patient's presentation was determined to be consistent with cognitive symptoms with non-neurodegenerative multifactorial aetiologies, including underlying medical conditions, fluctuating anxiety and medication side-effects, which could exacerbate cognitive impairment. There were no features of ‘internal inconsistency’ that suggested a contribution of a functional cognitive disorder to her subjective cognitive complaints.^15^ We discussed the diagnosis with the patient and provided a detailed explanation regarding the correlation between her cognitive symptoms, such as concentration and multitasking difficulties, and the level of the stress she was experiencing. We provided reassurance that there was no evidence of a major neurocognitive disorder due to a neurodegenerative condition. We emphasised the importance of anxiety treatment to address her cognitive complaints.

It was recommended that she taper and discontinue alprazolam because of its potential contribution to her cognitive complaints. Sleep hygiene practices were reviewed, and we discussed the use of herbal supplementation, such as with melatonin, to help with sleep initiation. We recommended a trial of sertraline, to address her anxiety and panic symptoms, given our suspicion that these symptoms were contributing to her distress regarding her poor short-term memory, attention and concentration problems. Sertraline was chosen as it had the least side-effect potential of the medications in its class in this case. We also recommended cognitive–behavioural therapy (CBT), mindfulness and non-pharmacological strategies to manage the patient's anxious distress.

The Brain Medicine Clinic provided follow-up care to this patient 2 months after her initial consultation. She had initiated sertraline but had not yet accessed CBT sessions. We practised distraction strategies, including focusing on an object or a sensory experience, and breathing techniques to calm her panic attacks. In follow-up, we continued to monitor her cognition. By the patient's third visit, she reported no severe panic attacks and subjectively experienced improvement in her cognitive and daily function.

This patient received an integrated psychiatric and neurological assessment, resulting in her condition being understood through a neurobiopsychosocial formulation. Using this formulation, the patient was provided with a management strategy that addressed all aspects of her presentation, rather than having to seek care from a neurologist and psychiatrist separately, who likely would not have collaborated on the root cause of her complaints, leading to delays in diagnosis and management. The patient acknowledged that the comprehensive treatment plan stemming from the integrative model of care seemed to accelerate her recovery and improved her experience of healthcare system navigation.

### Case 2

A 63-year-old right-handed woman was referred to the Brain Medicine Clinic for a 16-year history of cognitive complaints. She had a medical history of hypertension, diabetes mellitus (type 2) and obstructive sleep apnoea. Her psychiatric history included an 18-year history of major depressive disorder (MDD) that was suboptimally treated owing to the patient's inability to adhere to treatment, and a diagnosis of post-traumatic stress disorder following a motor vehicle accident 15 years ago. Her cognitive symptoms began immediately after the motor vehicle accident. In that accident, the patient sustained multiple injuries to her back and limbs, which led to chronic pain and disability. She was not known to have incurred a traumatic brain injury at the time. The patient had been started on bupropion 450 mg and duloxetine 120 mg for treatment of depression 3 months prior to her presentation in the Brain Medicine Clinic. She was also prescribed prazosin 2 mg to treat nightmares relating to the accident. Her cognitive complaints were described to have had a progressive pattern and to have increased in intensity in the years prior to presentation, manifesting as difficulties in attention, concentration and word finding, and short-term memory loss. She was independent in all her basic and instrumental activities of daily living, although she noted that planning and multitasking took more effort than previously. The patient reported feeling quite anxious about her difficulties with her cognition, as she worried about her ongoing independence. She lived with her two daughters and her grandson, who helped her when needed.

Mental status examination revealed a depressed mood, anhedonia and the presence of auditory and visual hallucinations, which followed a hypnopompic pattern and rarely happened during the day. She had insight about her psychotic symptoms and did not find them distressing. Virtual cognitive testing done by telephone revealed a MMSE-Blind score of 21/22 and a MOCA-Blind score of 18/22, with her losing points on delayed recall. Her virtual neurological examination (conducted over a video teleconferencing platform) was unremarkable.^16^ A computed tomography scan of the brain, completed 3 months prior to the assessment, revealed an extra-axial mass at the junction of the tuberculum sellae and planum sphenoidale eccentric to the right side with mass effect. An MRI brain (performed without contrast) further characterised this mass as in keeping with a meningioma. There were no findings on imaging or physical examination consistent with increased intracranial pressure, and the imaging findings were thought to be incidental to the patient's presenting complaint. Given this patient's multiple medical comorbidities and the limited cognitive assessment, several differential diagnoses were considered, with the most likely diagnosis being cognitive impairment due to a primary psychiatric disorder (in this case MDD with psychotic features). Other less likely differential diagnoses were mild cognitive impairment (MCI) with multiple aetiologies, such as vascular or neurodegenerative causes.

We recommended continuing duloxetine but suggested that her family doctor reduce the dosage of bupropion, given the intracranial mass and the propensity of bupropion to lower seizure threshold. We recommended against initiating cognitive enhancing medication at this time, since there is no evidence for using such medication in MCI and we did not suspect a neurodegenerative aetiology for her symptoms. We did not suggest any medications for her hallucinations, as these symptoms were not causing any significant distress and could be monitored over time. We recommended CBT for her depressive symptoms. With the collaborative approach to this patient, we were able to reach the most likely diagnosis. More importantly, this clinic visit helped prevent potentially harmful interventions, namely by reducing the bupropion dose and not starting an antipsychotic medication, through utilising an integrated care lens.

## Discussion

The Brain Medicine Clinic assesses and treats patients whose chief complaint includes symptoms in two or more of the domains of ABC. For the initial assessment, patients are provided with joint consultation from a neurologist and psychiatrist. Patients are initially seen by Brain Medicine fellows and are reviewed in interdisciplinary rounds involving neurology and psychiatry input. Cases are formulated utilising a neurobiopsychosocial model. Since assessment by both neurology and psychiatry co-occurs, patients seen in the clinic receive an integrated opinion about their diagnosis and management plan. As demonstrated by the cases described above, the Brain Medicine Clinic can provide patients with definitive diagnoses, reassurance about overlapping symptoms and a time-saving process by eliminating the need to see different specialists across separate visits. Additionally, it offers comprehensive, individualised treatment plans that can include medications for both psychiatric and neurological symptoms; initiation of cognitive and supportive psychotherapy; referral to further investigations; connection to other healthcare services and interprofessional healthcare team members; and prevention of unnecessary interventions that could result in harm. These cases demonstrate the proof of concept that using a neurobiopyschosocial approach facilitates patient-centred clinical care, bringing an element of precision medicine into the care of patients with complex brain disorders.^17^

Both cases described in this report provide qualitative support for the utility of the integrated, interdisciplinary assessment provided by the Brain Medicine Clinic. Had both cases been referred to a neurologist alone, there likely would have been delays to full diagnosis and treatment (and a longer period of symptoms related to cognition) due to the contribution of anxiety and affective complaints to the cognitive symptoms of concern. Moreover, in Case 2, it is probable that a neurologist would have had difficulty parsing the nuances of the patient's psychosis, possibly leading to a referral to a psychiatrist for consideration of antipsychotic medication, which carries an increased risk of mortality in ageing individuals.^13,18^ Conversely, if these cases had been referred to a psychiatrist alone, the treating specialist would likely have been uncomfortable definitively relating the cognitive symptoms to psychiatric symptoms without neurological input into interpretation of the neurological exam, brain imaging and cognitive testing. The two cases are illustrative of how the Brain Medicine Clinic provides appropriate individualised integrated treatment plans: although there are commonalities in how the underlying psychiatric symptoms were addressed, the interdisciplinary assessment highlights distinctions in each case that led to differences in recommendations.

Collaborative models of care can decrease stigma for patients experiencing psychiatric symptoms and increase communication between medical specialists.^3,5,13^ Many patients with neurological illnesses manifest psychiatric symptoms with impairment in their affect, cognition, perception or thought.^19,20^ These comorbidities complicate the diagnosis and treatment of common conditions such as Parkinson's disease, Alzheimer's disease and stroke.^3,19^ The most common psychiatric symptoms in neurological conditions such as Parkinson's disease are depression, apathy and anxiety, although 40–50% of patients develop delusions or hallucinations.^6^ For example, in a memory clinic, depression, psychosis and anxiety were all found to be symptoms of MCI.^21,22^ Indeed, both cases presented here demonstrate that experiences of disturbance in cognition tend to precipitate anxiety because of patients’ fear that their diminished cognitive abilities will leave them ill-equipped to cope with challenges that they may face in life. Moreover, the presence of anxious thoughts can contribute to subcortical dysfunction, further perpetuating underlying attentional and short-term memory concerns. These cases support the use of psychological interventions in complex brain disorders:^17^ these treatment modalities provide relief from neuropsychiatric symptoms and consequently can improve cognition by removing the confounding effects of symptoms such as depression and anxiety. Failing to work together in an interdisciplinary clinic might result in undiagnosed and untreated conditions and increase the burden of illness on patients, their family members and the wider healthcare system.

As illustrated by the two case examples described in this report, the Brain Medicine Clinic offers a highly practical and beneficial care model for individuals experiencing complex brain disorders. The interdisciplinary, integrated assessments and treatments available through this clinic expand the abilities of each type of involved ‘brain’ specialist beyond the service provision that could be delivered by either neurological or mental health services alone.

### Future development

Future directions for the Brain Medicine Clinic include integrating more ‘brain’ specialists, including physical medicine and rehabilitation, geriatric medicine and neurosurgery, to expand the interdisciplinary nature of the assessment and treatment modalities to achieve the most comprehensive patient care.

## Data Availability

Data availability is not applicable to this article as no new data were created or analysed in this study.

## Footnotes

a Brain medicine involves localising these categories of symptoms to both anatomical and functional (e.g. neurocircuitry, neurotransmitter) dysfunction. We refer to Arciniegas et al's localising framework, which includes perceptual disturbances under ‘behavioural alterations’ due to brain dysfunction.^14^
